# LinRace: single cell lineage reconstruction using paired lineage barcode and gene expression data

**DOI:** 10.21203/rs.3.rs-2777818/v1

**Published:** 2023-05-24

**Authors:** Xinhai Pan, Hechen Li, Pranav Putta, Xiuwei Zhang

**Affiliations:** 1 School of Computational Science and Engineering, Georgia Institute of Technology, Atlanta GA 30332, USA

**Keywords:** CRISPR/Cas9 genome editing, single cell lineage reconstruction, Neighbor Joining, Maximum likelihood

## Abstract

Understanding how single cells divide and differentiate into different cell types in developed organs is one of the major tasks of developmental and stem cell biology. Recently, lineage tracing technology using CRISPR/Cas9 genome editing have enabled simultaneous readouts of gene expressions and lineage barcodes in single cells, which allows for the reconstruction of the cell division tree, and even the detection of cell types and differentiation trajectories at the whole organism level. While most state-of-the-art methods for lineage reconstruction utilize only the lineage barcode data, methods that incorporate gene expression data are emerging, aiming to improve the accuracy of lineage reconstruction. However, effectively incorporating the gene expression data requires a reasonable model on how gene expression data changes along generations of divisions. Here, we present LinRace (**Lin**eage **R**econstruction with **a**symmetric **ce**ll division model), a method that integrates the lineage barcode and gene expression data using the asymmetric cell division model and infers cell lineage under a framework combining Neighbor Joining and maximum-likelihood heuristics. On both simulated and real data, LinRace outputs more accurate cell division trees than existing methods for lineage reconstruction. Moreover, LinRace can output the cell states (cell types) of ancestral cells, which is rarely performed with existing lineage reconstruction methods. The information on ancestral cells can be used to analyze how a progenitor cell generates a large population of cells with various functionalities. LinRace is available at: https://github.com/ZhangLabGT/LinRace.

## 1 Introduction

Understanding how cells divide and differentiate into various cell types is a fundamental problem in developmental biology. *Lineage tracing* technology which traces cell divisions using a “recorder” is the most widely used technique to study the developmental histories of cells, while traditional lineage tracing technologies can only work with a limited number of cells with low resolution [16]. Recently, sequencing-based lineage tracing methods (e.g. using CRISPR/Cas9 genome editing) have enabled the simultaneous recording of the clonal relationships of single cells alongside the transcriptomes [34] for up to thousands of cells. Such methods utilize lineage recorders, which are exogenous DNA sequences integrated into the genome. Even though there are different ways of designing the lineage recorder [1,2,3,13,20,25,31,32], the common idea is to introduce changes at the target sites (the location to which the Cas9 protein binds to induce mutations) on the lineage recorder which accumulates through generations of cell divisions. Finally, the recorders are sequenced together with the transcriptome of every single cell, resulting in the “lineage barcode” data. Each target site corresponds to a character in the barcode. The barcode data are used to reconstruct the cell division tree, which is also called the cell lineage tree in this paper. The reconstructed cell division tree can shed light on the developmental process that can not be directly measured.

However, inferring the cell division tree of a massive number of cells is a challenging problem. In addition to the computational complexity of inferring the lineage tree itself, the quality of the barcode data has posed further challenge to this problem [30]. First, the number of target sites, which is the length of the string used for tree reconstruction, is usually small (less than 10 [36]); Second, dropouts in the CRISPR/Cas9 induced lineage barcode data can cause missing information in the data. There are two types of dropouts: one is called *excision dropout*, or collapse dropout [7], result in the loss of consecutive target sites in between two simultaneous mutations (in this case, the mutations are deletions in the barcode). The other type of dropout is due to the limited capture efficiency of the sequencing experiment, where the barcodes of certain cells are not profiled. Finally, the biased distribution of mutations across the barcode and the number of mutations, represented by the *mutation rate* parameter, may not be optimal for reconstructing the cell division tree. Is it shown that the distribution of mutations across the barcode is not uniform, but rather biased towards certain target sites [29,24]. The mutation rate, which represents the efficiency of mutations being induced in the barcode, has a major impact on the potential to successfully inferring the cell division lineages from the data. However, current experimental technologies do not guarantee that the mutations occur at a rate that allows the tracing of every cell division event.

Various methods for tree inference from lineage barcodes have been developed. Recently, a DREAM challenge was held to gather the community effort to compare the state-of-the-art lineage tree inference methods [9]. Among the benchmarked algorithms the best performers are: DCLEAR [10], a distance-based method that first calculates the pairwise distance between cells and then reconstructs the cell lineage using bottom-up (agglomerative) algorithms such as Neighbor Joining (NJ) [28] or FastME [17]; Cassiopeia [12], a parsimony based method that aims at minimizing the number of mutations occurred on the reconstructed lineage tree. However, current methods for cell lineage tree reconstruction do not provide satisfying results using barcode data [9,24,29]. Moreover, due to the short barcode length and dropouts, a number of cells can have the same barcode, and the reconstructed lineage trees tend to have low depth (maximum path length from the root to a leaf node) and few internal nodes (with some nodes having large degrees) even using a perfect method. More recently, methods that combine lineage barcode and gene expression data are proposed, aiming to further improve the accuracy of cell lineage reconstruction. LinTIMaT [37] develops a combined likelihood function and uses a local search framework to search for the tree with the maximum likelihood. Integrating paired gene expression to infer the cell lineage tree can potentially refine the reconstruction, however, despite that the paired data should theoretically provide more information than barcodes alone, LinTIMaT did not beat methods that use only the lineage barcode data according to previous comparisons on synthetic datasets [24].

The key to combining the lineage barcode and gene expression data is to model the relationship between the two types of data, *i.e.*, how the gene expression of cells changes along with the barcode data during cell divisions. However, it is still an open question how the cell’s transcriptome changes during cell division. A simple assumption is to assume that cells that have similar transcriptomes should locate close in the cell division tree, *i.e.*, they should have similar barcodes. This is the assumption used in LinTIMaT. However, in a few recent papers that present paired single-cell lineage barcode and gene expression data, it is observed that, in a tree reconstructed using the barcode data, although a proportion of cells with the same cell state located in the same subtree, it is remarkable that some cells of the same cell state are located in different subtrees, and the same subtree can have multiple cell types [3,25]. We call this phenomenon the “partial consistency between transcriptome similarity and barcode similarity”.

The *asymmetric cell division model* has been shown to be able to account for the “partial consistency between transcriptome similarity and barcode similarity” [24]. It is commonly considered that cells can divide in a symmetric or asymmetric manner [15,21]. A symmetric cell division gives rise to daughter cells with the same cell state as the parent cells. During an asymmetric cell division, one daughter cell keeps the parent’s cell state, and the other one differentiates into a future cell state according to the cell state tree. For a given cell, there is a probability with which it divides asymmetrically, termed as the *asymmetric division rate*, denoted by *p*_*a*_. It has been shown that this probabilistic asymmetric cell division model leads to realistic paired single-cell lineage barcode and gene expression data.

To address these problems, we present LinRace, a new method that combines the lineage barcode and gene expression data to infer cell division trees, based on a joint Neighbor Joining (NJ) and maximum-likelihood framework. The asymmetric cell division model is used in LinRace to infer the states of ancestral cells and to calculate the likelihood function, thus incorporating the relationship between lineage barcode and gene expression data in a realistic way. On both simulated and real datasets, LinRace consistently outperforms the state-of-the-art methods according to multiple metrics. We show that the use of gene expression data in LinRace helps to improve the lineage tree reconstruction accuracy compared to methods that use only the lineage barcode data (Cassiopeia and DCLEAR). We also show that LinRace achieves better performances than the existing method that also uses gene expression data (LinTIMaT) while improving computational efficiency. We also propose a new metric, *reconstruction potential*, to analyze how LinRace performs compared to a theoretically “perfect” algorithm given the same input data. Moreover, we also demonstrate that when applied to large-scale real datasets, LinRace uncovers more detailed local lineage structures compared to LinTIMaT, as well as ancestral state information and state-lineage relationships that are consistent with observations from real data.

## 2 Results

### 2.1 Overview of LinRace

**Fig. 1.**
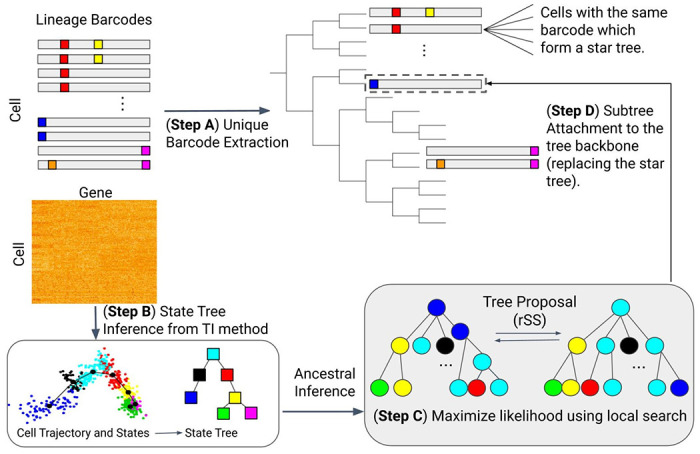
Overview of LinRace. **Step A** For barcode data, we extract the unique barcodes, and then perform Neighbor Joining to obtain the tree backbone, where each leaf represent a unique barcode shared by some cells. **Step B** For the gene expression data, we use kmeans on PCA reduced dimensions to infer the cell states, and then use Slingshot[33] to infer the state trajectories which is then used to infer the ancestral states. **Step C** For each group of cells of the same barcode, we use a maximum likelihood + local search framework to find the subtree topologies of the cells. **Step D** The final output tree is obtained by combining the subtrees at their specific leaves on the tree backbone. Nodes in the trees that are illustrated by square represent cell states (or cell types), and those illustrated using circles represent cells, and the color of each circle node represent the state of the cell.

LinRace is motivated by the fact that currently available lineage barcode data cannot label each cell with a unique barcode, and can contain large sub-groups of cells with the same barcode. For example, the embryo2 dataset in the mouse lineage tracing system [3] has 19019 cells but only 2788 unique barcodes. Among all the barcodes, 1929 of the 2788 barcodes uniquely label one cell; 330 of the 2788 barcodes are shared by two cells. Supplementary Fig. 1a shows the number of cells sharing each barcode, and only the top 50 of the barcodes which are shared by the largest number of cells are shown. 86.4% of the cells share the same barcode with at least two other cells. The barcode data alone does not allow the inference of trees for the cells with the same barcode. LinRace aims to infer the cell lineage tree using paired lineage barcodes and gene expression data, where every single cell has its corresponding lineage barcode and gene expression data. The overview of LinRace is shown in Fig. 1a.

First, from the barcode data of all the cells, we extract the unique barcodes and build a tree of these barcodes (Fig. 1 Step A) using the neighbor joining [28] tree reconstruction method. We then obtain a tree where each leaf node represents a unique barcode and can correspond to multiple cells with the same barcode. We denote this tree by ${𝒯}_{0}$. Then we use the single-cell gene expression data to refine the tree ${𝒯}_{0}$. We consider that there exists an underlying cell state transition mechanism that can be represented by a *cell state tree*, following which different cell types emerge during the cell division processes. This cell state tree can be inferred from the single cell gene expression data using trajectory inference methods [27,33,35] (Fig. 1 Step B). The cell state tree along with the *asymmetric cell division* model together model the relationship between lineage barcode and the gene expression data and allow for the inference of cell states for ancestral cells, as well as the design of the likelihood function used in Step C of Fig. 1. Details on the motivation for using the asymmetric division model are in Methods. Then, for each leaf in ${𝒯}_{0}$ which corresponds to one unique barcode and potentially many cells, we use a novel likelihood function (Methods) to find the best bifurcating tree (which means every internal node has exactly two children nodes) that maximizes this likelihood (Fig. 1 Step C). This likelihood is based on the asymmetric cell division model and the cell state tree. Finally, we attach the subtrees inferred in Step C to the lineage tree inferred from the barcode data $\left({𝒯}_{0}\right)$ to get the complete lineage of single cells (Fig. 1 Step D).

### 2.2 LinRace outperforms existing methods on synthetic datasets under various settings

We first test LinRace’s tree reconstruction performance against baseline methods using simulated data. We use TedSim [24] to generate simulated paired single-cell gene expression and lineage barcode data, which is the only existing simulator that generates such paired data with a ground truth cell division tree. We compare the results of LinRace with the state-of-the-art lineage tree reconstruction methods, including Cassiopeia-greedy and Cassiopeia-hybrid [12] (a parsimony-based method), and DCLEAR-kmer [10] (a distance-based method), which use only the barcode data, and LinTIMaT [37], a method based on the combined likelihood of gene expression and lineage barcode that use both types of data.

To obtain a comprehensive picture of the performances of the methods, we vary major parameters when generating the simulated data: (1) Mutation rate *μ*, the probability that a mutation (insertion or deletion) is induced per target site per cell division. Different mutation rates can result in barcode data with different quality, and it has been shown that the performance of lineage tree reconstruction methods using only the barcode data is affected by the mutation rate [24,29]. A range of [0.01, 0.3] was used in previous work [29], and we used a similar range which is [0.05, 0.4]. (2) With or without dropout. Although real data contains a significant amount of dropouts, comparison of results on data with and without dropouts can show the effect of dropouts on each method; (3) Number of cells. The complexity of the lineage tree reconstruction problem increases with the number of cells. We generated datasets with 1024 and 4096 cells. For each combination of parameters, we generated 10 datasets with 10 random seeds. More parameter settings on data simulation and the simulation process are in Methods.

The software versions and parameters used for the baseline algorithms are in Methods. For LinRace, the major hyperparameters are $\lambda_{1}$ and $\lambda_{2}$, which are weights for different terms in the likelihood function (Method). we use the default settings, $\lambda_{1}=10$ and $\lambda_{2}=1$, for all datasets. Although we provide default values for $\lambda_{1}$ and $\lambda_{2}$, we show that LinRace is not sensitive to changes in these parameters. In Supplementary Fig. 2, we show the performances of LinRace are similar under various parameter settings for $\lambda_{1}$ and $\lambda_{2}$. Other parameters used for LinRace are specified in Methods.

As the states of cells and the cell state tree inferred in Fig. 1 Step B are used to infer the states of ancestral cells and calculate the likelihood (Fig. 1 Step C), the accuracy of the inferred cell states and cell state tree can affect the lineage tree reconstruction accuracy of LinRace. With the ground truth cell states and cell state tree provided by TedSim, we can investigate the effect of cell state and cell state tree inference on the final performance of LinRace. To do this, we run LinRace in two modes: (1) using the ground truth cell states and the cell state tree, this mode is denoted as LinRace-TST (True State Tree); (2) using inferred cell states and the cell state tree, and this mode is denoted as LinRace-IST (Inferred State Tree). We use Slingshot [33] to infer cell states and the cell state tree from the gene expression data. As Slingshot does not infer the direction of trajectories, it is a common practice to assign a root cell so that we obtain a directed trajectory. We randomly selected a cell from the root cell state in the true cell state tree to provide this cell to Slingshot as the root cell.

**Fig. 2.**
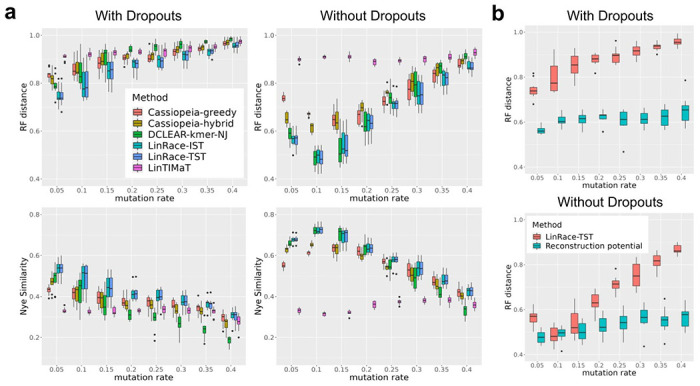
Benchmarking results for lineage reconstruction methods on TedSim simulated datasets. **a** Comparisons of LinRace (LinRace-IST and LinRace-TST) and other methods on TedSim simulated datasets using RF distance and Nye similarity. Both RF distance and Nye similarity have the range of [0, 1]. For both RF distance, lower is better, and for Nye similarity, higher values indicate the better performance. The detailed descriptions for simulation and method settings can be found in Methods. **b** Comparison of the reconstruction potential and LinRace-TST performances. The data used to calculate the reconstruction potential and the RF distance of LinRace-TST reconstructed trees are the same data from **a**.

To evaluate the quality of reconstructed lineage trees, we use the RF (Robinson-Foulds) distance [26] and Nye Similarity score [22] to quantify the distance or similarity between the reconstructed lineage tree and the true lineage tree (Methods). For RF distance, lower is better, and for the Nye Similarity, higher is better. The benchmarking results are shown in Fig. 2 and a detailed description of the simulation settings and methods setup can be found in Supplementary Note 2.

The results of LinRace-TST, LinRace-IST and baseline methods on all simulated datasets with 1024 cells are shown in Fig. 2a. On the datasets with 4096 cells, the Nye similarity is too slow to run, so we mainly discuss the results on 1024 cells (the RF distance results for 4096 cells are shown in Supplementary Fig. 3). One can observe that most methods show a similar trend when the mutation rate changes, except for LinTIMaT, which shows much smaller fluctuations and worse performances when there are no dropouts. We think the intractable size of tree space for 1024 leaves makes it extremely difficult to run a tree local search for LinTIMaT. For the other methods, the optimal mutation rate for lineage tree reconstruction is around 0.1-0.15 without the presence of dropouts. With dropouts, all methods perform worse when the mutation rate increases. The trends of the performance of LinRace with the increase of mutation rate are expected and are consistent with the observation in [29]. When the mutation rate is extremely small, the barcodes are not distinct enough to infer the tree, and when the mutation rate is too large, more excision dropouts occur and the quality of barcode data decreases, therefore, the performances decrease even further.

The two modes of LinRace, LinRace-TST and LinRace-IST, have similar overall best performances shown in Fig. 2a. This similarity in performance indicates that LinRace is robust to inference errors in the cell states and cell state tree, which confirms the applicability of LinRace on real datasets, where true cell states and cell state trees are not available. Since Slingshot was run on each dataset obtained with a given parameter setting and random seed separately, the inferred cell state tree for each dataset can be different, as visualized in Supplementary Fig. 4. Additionally, when inferring cell states, the ground truth number of cell states is usually unknown. In our evaluation, we used a generic parameter of 7 for the number of states, which is the same as the number used for real data (Methods). However, it should be noted that the ground truth number of cell states for the simulated data set is 52, as shown in Supplementary Fig. 4. Despite this difference, our results demonstrate that LinRace is robust to the number of cell states, as evidenced by the similar performances of LinRace-TST and LinRace-IST.

When the barcode data quality is good, that is, with no dropouts and with optimal mutation rate (from 0.1 to 0.2), DCLEAR-kmer, which uses only barcode data, achieves comparable or slightly better (Fig. 2a) performance as LinRace. However, we emphasize that current lineage barcode data is far from this quality, thus incorporating the gene expression data in an appropriate way is necessary. We also observed similar patterns for the datasets with 4096 cells (Supplementary Fig. 3).

Under different conditions (mutation rate, presence of dropout, number of target sites), the quality of the lineage barcode data will limit how well a method can reconstruct cell lineage using only barcode data. In order to better visualize how the quality of the lineage tracing process changes, we propose a *reconstruction potential* score (see Methods), which gives an idea about how well the lineage tracing process labels each cell division event on the true lineage. For every split on the true lineage tree (an edge separating the leaf cells into two sets), we calculate a score: 0 is returned if a unique mutation separates two sides of the edge; 1 otherwise. Then, the sum of the scores is divided by the number of edges. Therefore, the reconstruction potential ranges from 0 to 1(0 means all edges are not detectable and vice versa) allowing us to directly compare the values of reconstruction potential with the RF distance of the lineage reconstruction methods like LinRace. From the results in Fig. 2b, we can see that dropouts not only affect the absolute RF distance values for LinRace but also the relative values between reconstruction potential and RF distance of LinRace. LinRace is able to get close to the reconstruction potential when the barcode data is good (μ=0.05−0.15 without dropouts), which means that the number of correctly inferred splits of LinRace is on par with the number of detectable splits on the true tree. The RF distance of LinRace can sometimes (μ=0.1 without dropouts) exceed the reconstruction potential, which can be potentially caused by local structures inferred from the gene expression data.

#### Computational efficiency analysis

The computational efficiency of tree reconstruction algorithms are also beyond significance. The computational cost of calculating the likelihood as well as the size of the tree space increases super-exponentially with the number of input cells. Therefore, most state-of-the-art methods such as DCLEAR and Cassiopeia use greedy heuristics to enable efficient tree reconstruction.

In LinRace, we perform a local search on the cells with the exact same barcode, therefore for one dataset, LinRace performs multiple local search processes on different numbers of leaves. The growth of the size of the tree space can be characterized using the Catalan numbers [5]. The fast growth rate of the tree space makes it impossible to perform an exhaustive tree search on even 50 leaves. To make our tree search process efficient and find the optimal subtree structure for different neighborhood sizes under one tree backbone, we adopt a dynamic local search strategy: for each group of cells, we determine the number of iterations based on the number of input cells to improve the efficiency of the algorithm.

Comparing LinRace with other state-of-the-art methods (Supplementary Fig. 5), we can show that LinRace achieves much faster running time than other local search-based methods such as LinTIMaT. Built upon Neighbor Joining, LinRace achieves similar or lower running time as the other heuristics, such as Cassiopeia-greedy and DCLEAR, when the number of cells is small. When the number of cells increases, LinRace runs more local searches on larger subtrees, which will increase its running time.

### 2.3 Evaluating LinRace and baseline methods on real C. elegans dataset

The ideal scenario to evaluate lineage tree reconstruction methods is to have the following information: the gene expression data and barcode data for single cells, and the ground truth cell lineage tree. While it is rare for experimental data to have ground truth cell lineage trees, *Caenorhabditis elegans* (*C. elegans*) is one of the few species that have the exact cell lineage resolved [23] with single-cell gene expression data measured. To obtain paired single-cell gene expression and barcode data for this system, we adopt a strategy used in [37] to simulate the barcode data from the known lineage tree. Therefore, we combine the measured gene expression data of *C. elegans* and simulated lineage barcode data from TedSim using the true lineage tree. We then apply different lineage reconstruction methods and compare the reconstructed lineage trees with the ground truth tree. When simulating the barcode data, we again vary the mutation rate and the existence of dropouts.

**Fig. 3.**
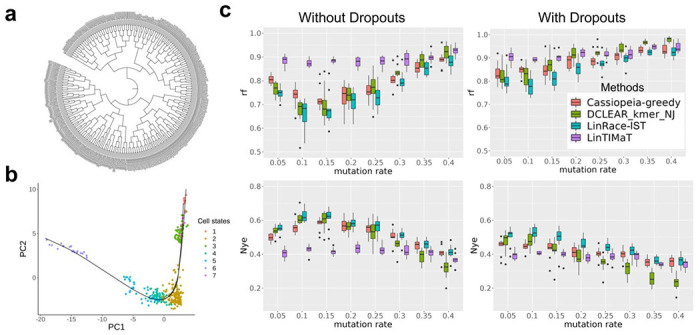
Results for tree reconstruction methods on real C.elegans datasets. **a** The exact true lineage of C.elegans at L1 larvae stage. There are 363 profiled cells on the tree while the original L1 larvae lineage has 668 cells. The tree pruning is done using the ape package in R and the clonal relations between cells are preserved. The tree is visualized using iTOL[18]. **b** 2-D PCA visualization of the gene expression of the C.elegans data. After PCA, the first 20 PCs are used for kmeans clustering and Slingshot to determine cell states and state trajectories (cell state tree). Detailed description of the processing steps for the gene expression data can be found in the Supplementary Note 2. **c** Benchmarking result on the C.elegans dataset with simulated barcodes. The methods are tested for varying mutation rate and dropout effect. Two metrics, RF distance and Nye similarities are used for the benchmark.

The dataset is obtained from Liu *et al.* [19], who profiled the gene expression lineage of 93 genes in 363 specific cells from L1 stage larvae. They used knowledge of the cell number, morphology of the cell nuclei, and their relative position with respect to each other to develop an automatic method to identify specific cells in confocal images of worms expressing a fluorescent reporter, and then measure expression in specific cell nuclei. The Newick format of the true lineage of the L1 larvae is obtained from CeLaVi [29] by Salvador-Martinez *et al.* which is then trimmed to the profiled 363 cells in the dataset (see Fig. 3a). From the visualization of reduced dimensions in Fig. 3b, we can see that the inferred trajectory is able to connect the cell states and forms a continuous manifold. The inferred cell state tree (shown in Supplementary Note 2) from Slingshot [33] is used to calculate the state transition likelihood in LinRace.

From the results in Fig. 3c, we can see that LinRace outperforms the state-of-the-art methods consistently for varying mutation rates, with or without dropouts. The overall results are consistent with the benchmarking results on simulated datasets. These results not only show the advantages of LinRace over baseline methods on real data but also confirm that LinRace does not need a true cell state tree to be effective. As long as the trajectory inference method captures the relative local relations between cell states, our likelihood function can effectively evaluate a candidate lineage tree based on the raw expression and state transitions. Comparing the performances of the methods with and without dropouts, we can see that LinRace outperforms other methods even more on datasets with dropouts, which indicates that integrated methods like LinRace are able to utilize the gene expression data to compensate for the loss of information caused by dropouts in barcode data. On the other hand, despite using gene expression data, LinTIMaT does not perform better than the two barcode-based methods with dropouts, DCLEAR and Cassiopeia. The reason can be two-fold: first, LinTIMaT runs local search on the whole lineage tree which allows it to explore only a small proportion of the search space, as optimizing this tree as a whole is an NP-hard problem [4]; second, the design of their likelihood function is based on an over-simplified assumption on the relationship between gene expression and barcode data.

### 2.4 LinRace reveals ancestral state transitions of zebrafish brain cells

In most studies that present jointly profiled scRNA-seq and lineage barcode data [1,2,3,13,20,25,31,32], the barcode data and the gene expression data are processed and analyzed separately, where the barcode data is used for building the cell lineage and the gene expression data is used to infer the cell types (Supplementary Fig. 6). Due to the poor quality of the barcode data, the lineage tree tends to have a relatively low resolution which is reflected by the shallow depth and the small number of internal nodes. Hundreds of cells of different cell types can be connected to the same node and their relative clonal relationships are unknown.

In this section, we show that LinRace can be used to obtain cell lineage trees with better local resolution than the state-of-the-art methods, as well as the cell states of ancestral cells, which is a unique function of LinRace. The finer local structure of the inferred lineage tree inferred by LinRace together with the cell states allows us to identify the location of symmetric and asymmetric divisions in the reconstructed lineage and obtain a picture of how cell types are formed during cell divisions.

**Fig. 4.**
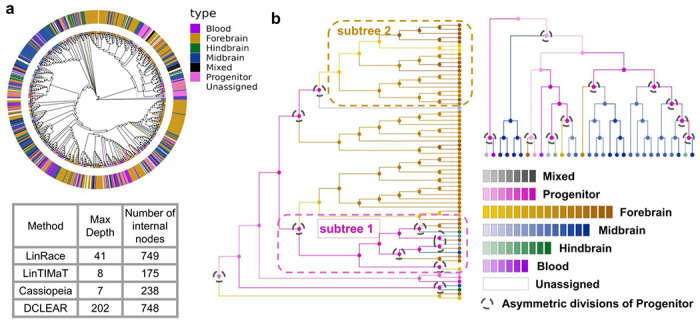
Reconstructed trees of ZF1-F3 sample (750 cells) from scGESTAULT datasets in [25]. **a** LinRace reconstructed tree. The outer ring represent major cell type assignments and the inner colors on edges show detailed intermediate cell type assignments. The statistics of the reconstructed trees for different methods are also shown, and the max depth means the maximum total edge length to go from the root to any leaf cell. **b** A detailed look of two GES in LinRace reconstructed tree with inferred ancestral states. In the left GES, Subtree 1 shows a subtree of progenitor’s self-renewal and subtree 2 shows a subtree of differentiated Forebrain population. Black dashed circles denote asymmetric divisions of progenitor cells. The annotated leaf cell states are from the original data paper, and the ancestral states of all hidden nodes are inferred using LinRace. Detailed description of the processing steps for the gene expression data are in the Supplementary Note 2.

We use a zebrafish brain dataset from scGESTAULT[25] and reconstruct the cell lineage using LinRace and other baseline methods (Fig. 4a and Supplementary Fig. 7). We are not able to provide quantitative accuracy of the reconstructed trees as there does not exist a ground true lineage tree. Instead, we compare the resolutions of the trees using the depth and number of internal nodes of the reconstructed trees. From Supplementary Fig. 7a, we see that the LinRace reconstructed lineage tree has more depth and inferred internal nodes than the Cassiopeia tree and LinTIMaT tree. For DCLEAR which also infers a bifurcating tree, the local splits between cells with the same barcode is randomly decided which does not provide any biological insights, and its number of internal nodes is almost the same as that of LinRace but its depth is much higher means that the DCLEAR tree is much less balanced than the LinRace Tree (Supplementary Fig. 7).

Fig. 4b shows two Gene Expression Subtrees (GES) where the color of nodes (including both leaf nodes and ancestral nodes) represent the cell types of cells. We would like to note that LinRace use inferred clusters to run trajectory inference while the visualized trees are colored with annotated cell types from the paper. From Fig. 4b, we can see where some progenitor undergoes asymmetric division and differentiate into some functional cell type, which in turn divide symmetrically into a large subtree of that cell type Subtree 2 in Fig. 4b). We can also observe the case where the progenitor cell divides symmetrically to keep the sustainable activity of neurogenesis (Subtree 1 in Fig. 4b).

Using the likelihood function, LinRace can infer the cell type of every internal node and the reconstructed local binary structure reflects realistic division histories that explain the observed cell types and gene expression data. LinRace shows at which locations (black dashed circles) on the lineage tree the progenitor cells divide asymmetrically and eventually lead to a large population of functional cell types (a Forebrain population and a Midbrain population in Fig. 4b, respectively).

Finally, from the visualization of reconstructed cell lineage trees with different methods (Fig. 4a and Supplementary Fig. 7), we can see that compared to the other methods, LinRace is able to reconstruct a lineage tree with a realistic distribution of cell types, where while cells with the same cell types tend to be located together in the reconstructed lineage tree, the same cell type can appear in different subtrees, shows the “partial consistency between transcriptome similarity and barcode similarity” in real data. In the next section, we further show this phenomenon using another dataset of the mouse embryo. Further, we show how asymmetric divisions explain this phenomenon and the conjecture of varying cell differentiation speeds on different lineages.

### 2.5 LinRace helps to answer the sources of diverse cell types in the mouse embryo data

**Fig. 5.**
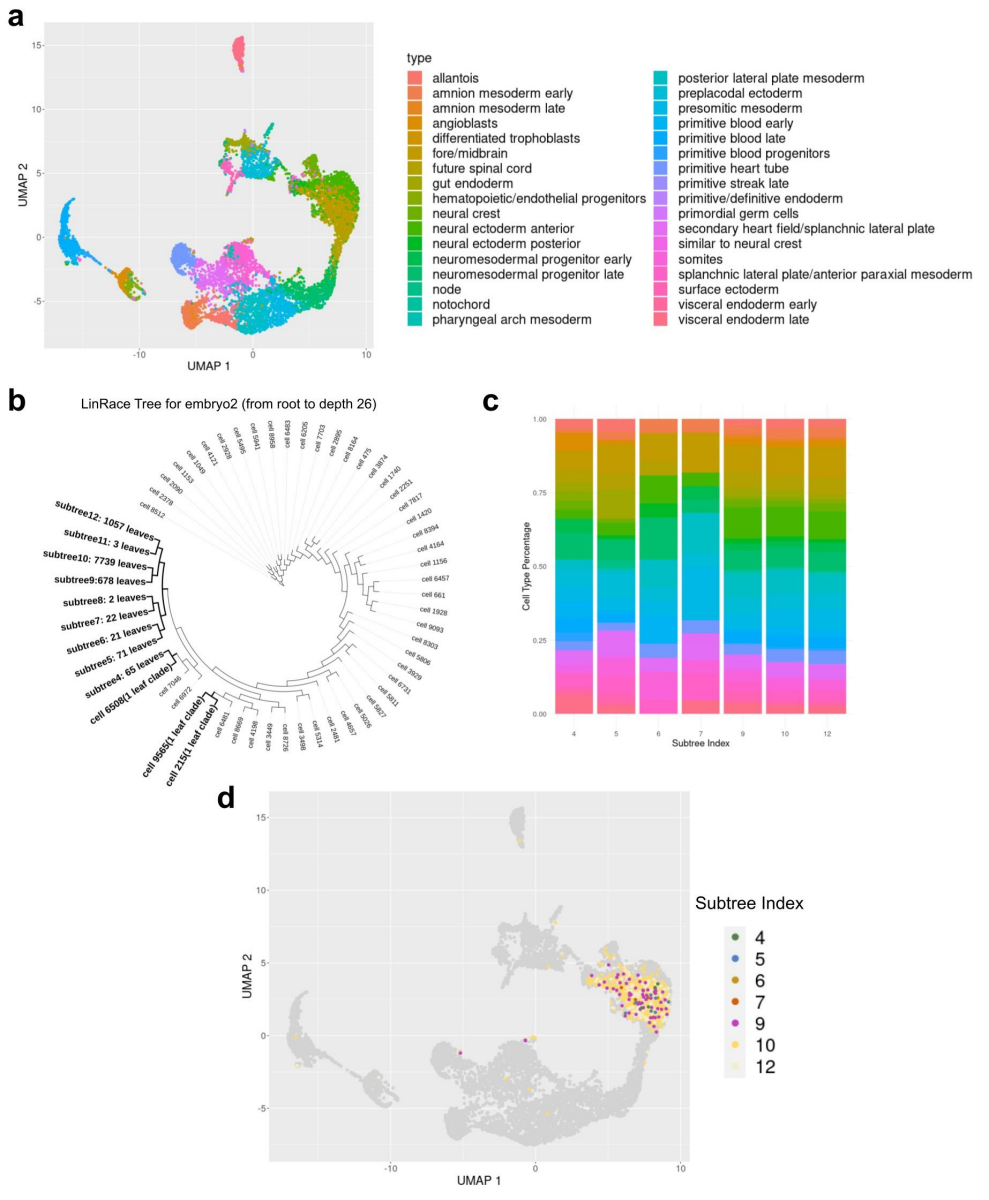
Visualizing inconsistencies between cell states and lineages. **a** UMAP visualization of the early mouse embryo dataset with cell type annotation from [3]. **b** LinRace reconstructed tree of the dataset. Only branches from root to *depth* = 26 is shown here, where the remaining subtrees are shown as leaf nodes, with the numbers of leaf cells attached to them. Three one-leaf clades are included which means they are leaf nodes at *dep* = 26. **c** Cell type compositions under different subtrees. The subtrees are obtained by cutting the whole lineage at the same depth (total edge length from the root to the cutting point.) 12 subtrees are obtained by cutting the lineage tree at *depth* = 26 and only subtrees have more than 10 leaves are shown here. The y-axis shows the percentage of each cell type in **a** under each subtree. **d** UMAP visualization of the Fore/Midbrain cell type by the clonal IDs. Only subtrees have more than 20 leaves are labelled and cells that do not belong to these subtrees are colored black and labelled as “0”.

We applied LinRace to an early mouse embryo dataset from Chan *et al* [3] to infer the cell division tree. The mouse embryo dataset contains 9707 cells of 34 annotated cell types from the authors (Fig. 5a). On this dataset, we not only analyze how the cell types are distributed over the cell division tree but also ask if any lineage signature exists in a cell’s gene expression profile.

We cut the reconstructed tree at depth 26 and obtained subtrees with their roots at a distance of 26 to the root of the complete tree (Fig. 5b). First, we investigate the cell type composition of the subtrees. The cell type composition of 7 subtrees with at least 10 leaves is shown in (Fig. 5c). Each subtree consists of multiple cell types (colors are consistent with the color legend of Fig. 5a), and the same cell type appears in different subtrees.

Next, we take the cells that belong to the same cell type (Fore/Midbrain) and look for differences that potentially exist between cells from different lineages but with the same cell type. We visualize the Fore/Midbrain cells labeled by their clonal IDs (same as the subtree IDs, Fig. 5d). In the UMAP space, cells from different clones are mixed, which demonstrates that there are no significant lineage-affiliated features in the reduced dimensions of the transcriptomic data. This observation supports the idea that the gene expressions of mature cell types are dominated by their functionality, not their lineage identity, which is also previously reported in [23].

Further, it is a common observation that in a scRNA-seq dataset, although all the cells are profiled at the same time, the cells can have different pseudotime representing varying stages of development or distinct cell states. In the context of the cell division tree that gives rise to the leaf cells, we conjecture that leaf cells at a later stage of development can originate from lineages with fast differentiation speed, potentially involving frequent asymmetric divisions, and leaf cells at an early stage of development can originate from lineages with slow differentiation speed.

## 3 Discussion

In this paper, we present LinRace, an integrated method that combines the lineage barcode and gene expression using the asymmetric cell division model. Compared to the state-of-the-art methods, LinRace has the following advantages: (1) Using the combined framework of NJ and maximum likelihood, LinRace outperforms the state-of-the-art methods on both simulated and real datasets; (2) LinRace proposes a novel likelihood function that takes into account mRNA counts, cell states and state transitions to find the lineage tree that best explains the observed gene expression data; (3) LinRace is able to infer the cell states of ancestral and provides insights on the process of generating new cell types; (4) LinRace performs local search on subtrees using a dynamic number of iterations based on the size of the subtrees which makes it computationally efficient compared to other tree search algorithms.

Reconstructing the cell division tree from lineage tracing barcodes is similar to the problem of inferring phylogenetic trees from genome data in the field of evolutionary biology [14]. However, the cell division tree reconstruction is even more challenging due to the much larger number of leaves (single cells) and the short lineage tracing barcodes with dropouts. Incorporating the single cell gene expression data is a promising direction, but developing such integrated methods requires modeling the relationships between the two modalities, the lineage barcode and the gene expression. LinRace adopts the asymmetric division model, which explains phenomena in real data, increases reconstruction accuracy, and allows for the estimation of ancestral cell states.

LinRace assumes that cell state transitions are irreversible, which is generally true under homeostatic conditions. However, reversible state transitions are also known to exist. To incorporate reversible transitions, it may be necessary to expand the definition of the cell state tree to a cell state network in future work. In addition to developing new computational methods, it is also important that the data quality is improved with advances in technology, in order to build the tree of cell division and cell differentiation with high accuracy.

## 4 Methods

### 4.1 The asymmetric division model accounts for cell state changes in the lineage tree

The asymmetric division of cells is a key process that leads to multiple cell types and different cell differentiation speeds on different clones on the cell lineage tree [6,21]. We show that the asymmetric division model can account for various scenarios of cell state change on a cell division tree reviewed in [34]. Moreover, it was shown to lead to realistic paired lineage barcode and gene expression data [24]. An asymmetric division results in two unequal daughter cells from the parent cell, one of which differentiates into a natural “next-state” (according to the cell state tree) while the other remains in the same cell state as the parent.

Wagner and Klein [34] reviewed hypothetical scenarios of restricted lineage trajectories unfolding on a state manifold of gene expressions. In the review, state convergence represents two or more distinct fate trajectories converging onto the same final position on a state manifold, and state divergence represents the reverse process where one trajectory bifurcates into two or more distinct fate trajectories. These seemingly contradictory scenarios can happen due to asymmetric cell divisions. Asymmetric divisions can cause one cell state to generate two distinct cell states, resulting in state divergence; and cells on distant lineage trees can also divide asymmetrically into the same cell state, resulting in state convergence. In LinRace, we account for both symmetric and asymmetric cell divisions in our likelihood function using the asymmetric division likelihood which estimates the prior probability of asymmetric and symmetric divisions based on the mutated states in the observed cells. We assume that state transitions on all parent-child cell edges are independent, and also consider the stochasticity of cells’ developmental speeds by varying the number of states traversed for each state transition. The dynamic and asymmetric cell divisions on the cell lineage tree are called the asymmetric division model in this paper. In LinRace, the asymmetric division model is also used in inferring ancestral cell states.

### 4.2 Reconstructing lineage backbone from the lineage barcode data

The lineage barcode of a cell is represented as a character vector of length equal to the number of target sites as designed by the CRISPR/Cas9 lineage recorder. Each character represents a state of the target site, which can be a mutated state, an unmutated state, or a dropout state. We use “0” to represent the unmutated state, and each unique non-zero character represents a unique mutation state, regardless of the position where the mutation is observed. The dropout state is denoted as the “-” character. For the barcode data, we assume that at the root of the cell lineage, an unedited DNA sequence (all unmutated states) is introduced. During cell divisions, unmutated states can potentially mutate and will never mutate again, except the dropout state. Given *N* cells and *M* targets, the lineage barcode data is a *N* × *M* matrix. For the barcode data, we assume that at the root of the cell lineage, an unedited DNA sequence is introduced. During cell divisions, unmutated target sites can potentially mutate and will never mutate back to the original state (except dropout state).

In LinRace, we utilize a Hamming distance-based NJ method to infer the “backbone lineage tree”. Since multiple cells can have the same barcode in the dataset, running Neighbor Joining on the *N* × *M* matrix will result in merging cells with the same barcode in some random order. With a given lineage barcode matrix, we first transform it into a *K* × *M* matrix where each row represents a unique barcode in the original data, and then Neighbor Joining is applied to the *K* × *M* matrix to get the lineage tree of unique barcodes (Fig. 1). We call this tree the reconstructed lineage backbone, or tree backbone (denoted by ${𝒯}_{0}$).

### 4.3 Inferring cell states for ancestral cells

To calculate the likelihood of a candidate tree based on the gene expression profiles of the leaves, we first need to infer the states of cells at ancestral nodes using the states of leaf nodes and the cell state tree that are inferred during Fig. 1 Step B. The inference of ancestral cell state follows the rule of asymmetric division. From leaves to the root of the candidate lineage tree, we consider the following cases: (1) If the daughter cells have the same cell state, their parent is assigned the same state, meaning the parent cell divides symmetrically. (2) If the daughter cells have different cell states, the parent cell will have the Most Recent Common Ancestor (MRCA) cell state based on the cell state tree. The ancestral cell states in trees in Fig. 1 Step C follow these two rules given the cell state tree learned from Step B. This ancestral state inference process allows one cell to divide into two cells with different cell states. If two daughter cell states belong to the same differentiation path (a path from the root state to a leaf state), the parent cell will be the same cell state as the earlier cell state between the two.

### 4.4 Finding subtree topology using a maximum likelihood method

Cells with identical barcode form a star tree in the initial NJ tree ${𝒯}_{0}$ (See Fig. 1). LinRace utilizes the gene expression data to learn the bifurcating tree topology of these cells. The learned bifurcating trees, termed Gene Expression Subtrees (GES), are then attached to the tree backbone to yield the full cell lineage tree. We design a maximum likelihood scoring function and local search strategy to find the GES.

#### Likelihood of a candidate lineage tree

We design a likelihood function to evaluate how well a candidate lineage tree explains the observed gene expression data. We use the ancestral state inference step mentioned above to determine the cell states of all nodes on the tree. Then, we can calculate the likelihood of the tree, which consists of three terms: *state transition likelihood, asymmetric division likelihood*, and *neighbor distance likelihood*. The first two terms use the cell state information of cells, and the last uses the gene expression profiles of the cells.

The *state transition likelihood* represents the probability of transitions between cell states on the edges of the lineage tree. We adopt the assumption that the state transition on each edge is independent of other transition events (this assumption is commonly used in phylogenetic tree reconstruction) so that we can write the state transition likelihood of a given lineage tree 𝒯=(E,V) as:

(1) $${ℒ}_{\varepsilon }\left(𝒯\right)=P\left(𝒮|𝒯\right)=\prod_{e\in E}P\left({𝒮}_{e_{2}}|{𝒮}_{e_{1}}\right)$$

where $e=\left(e_{1},e_{2}\right)$ represents an edge on the tree graph from node $e_{1}$ to $e_{2}$, 𝒮 denotes the cell state assignments of all cells, and ${𝒮}_{e_{1}}$ and ${𝒮}_{e_{2}}$ denotes the cell states of the two cells $e_{1}$ and $e_{2}$ connected by e∈E.

The state transitions of cells’ gene expressions are governed by an underlying developmental cell state tree, which is inferred from the gene expression data using Slingshot (Fig. 1 Step B). The cell state tree guides the cells to differentiate into certain future cell states irreversibly. For any two states on the cell state tree, there can exist at most one path(a sequence of connected, directed edges) that links these two states. Therefore, when the states of a pair of ancestor-descendant, denoted as $\left(S_{e_{1}},S_{e_{2}}\right)$ between two cells $\left(e_{1},e_{2}\right)$, the transfer probability $P\left({𝒮}_{e_{1}}|{𝒮}_{e_{2}}\right)$ is calculated as follows:

(2) $$P\left({𝒮}_{e_{2}}|{𝒮}_{e_{1}}\right)=P\left(𝒟\left(S_{e_{1}},S_{e_{2}}\right)\right)$$

where $𝒟\left(S_{e_{1}},S_{e_{2}}\right)$ represents the graph geodesic from $S_{e_{1}}$ to $S_{e_{2}}$ on the cell state tree. If $𝒟\left(S_{e_{1}},S_{e_{2}}\right)=+\inf$, a penalty of −50 is applied to the log likelihood. The probability for every distinct state transition is given as follows:

(3) $$P\left({𝒮}_{v}|{𝒮}_{u}\right)=\frac{\sum_{\left(u^{\prime },v^{\prime }\right)\in E}\;𝟙\left(𝒟\left({𝒮}_{u^{\prime }},{𝒮}_{v^{\prime }}\right)=𝒟\left({𝒮}_{u},{𝒮}_{v}\right)\geq 0\right)}{\sum_{\left(u^{\prime },v^{\prime }\right)\in E}\;𝟙\left(𝒟\left({𝒮}_{u^{\prime }},{𝒮}_{v^{\prime }}\right)\geq 0\right)}$$

The *asymmetric division likelihood* considers the asymmetric divisions in the lineage tree and it is defined as follows:

(4) $${ℒ}_{ad}\left(𝒯\right)=\prod_{\left(s_{ances},s_{1}\right),\left(s_{ances},s_{2}\right)\in 𝒯}P_{ad}\left(s_{1}==s_{2}\right),\;\text{where}\;P_{ad}\left(s_{1}==s_{2}\right)=\{\begin{array}{cc} 1-p_{a}, & s_{1}=s_{2}\\ p_{a}, & s_{1}\neq s_{2} \end{array}$$

where $s_{ances}$ is the state of a parent cell and $s_{1}$, $s_{2}$ are the states of the two descendant cells. The asymmetric division rate $p_{a}$ can be determined using prior knowledge or inferred from the fraction of observed asymmetric neighbors in the lineage tree.

For *neighbor distance likelihood*, we look at cells that are siblings (having the same parent node) on the candidate lineage tree, and use the transition probability from diffusion map [11] to evaluate if the two cells are locally connected on the developmental trajectories of the gene expression data. Even though in general when asymmetric division happens, the two daughter cells do not have the same cell states thus their transcriptomes are not very similar, we consider that many cells at the leaves of the lineage tree are terminal state cells, and asymmetric divisions are less prominent when more cells are at terminal states. We denote the set of measured cells by Ω. First, for all pairs of cells $\left(x_{i},x_{j}\right)$ in Ω, we calculate the pairwise distance of cells’ gene expression data using the radial basis function (RBF) kernel $K_{ij}=exp\left(\frac{-{\left\|x_{i}-x_{j}\right\|}^{2}}{2\sigma^{2}}\right)$. Then, the transition probability between any two cells can be calculated as follows:

(5) $$Z\left(x_{i}\right)=\sum_{x_{k}\in \Omega }K_{ik}\;\;\;\;\;\hat{Z}\left(x_{i}\right)=\sum_{x_{k}\in \Omega /x_{i}}\frac{K_{ik}}{Z\left(x_{i}\right)Z\left(x_{k}\right)}$$

(6) $$P_{nd}\left(x_{i},x_{j}\right)=\frac{1}{\hat{Z}\left(x_{i}\right)}\frac{K_{ij}}{Z\left(x_{i}\right)Z\left(x_{j}\right)}$$

and the neighbor distance likelihood can be calculated as:

$${ℒ}_{nd}\left(𝒯\right)=\prod_{x_{i},x_{j}\in \Omega ,\left(x_{i}\leftarrow u\to x_{j}\in 𝒯\right)}P_{nd}\left(x_{i},x_{j}\right)$$

Finally, the total likelihood is calculated as:

$$ℒ\left(𝒯\right)={ℒ}_{\varepsilon }\left(𝒯\right)+\lambda_{1}\cdot {ℒ}_{ad}\left(𝒯\right)+\lambda_{2}\cdot {ℒ}_{nd}\left(𝒯\right)$$

where $\lambda_{1}$ and $\lambda_{2}$ are hyperparameters.

To find the best GES based on our likelihood function, we utilize hill-climbing local search to search in the tree space (Fig. 1 Step C). In order to propose a new tree from a current tree, we adopted random Subtree Swapping (rSS), which is a derivative of the widely used random Subtree Pruning and Regrafting (rSPR) [8]. We randomly select two nodes on the current tree and prune the subtrees attached to the specific nodes. Then, we regraft either one of the pruned subtrees to the location of the other subtree. The advantage of rSS is that with an initialization of a bifurcating tree, this operation will not disrupt the binary property throughout the searching process. This move can result in most of the applicable topological changes of the tree so we use it as the search technique. At every iteration, a new tree is proposed which is one rSS move away from the current tree. Then, we evaluate the new tree with the likelihood function and compare it with the likelihood of the current tree. If the new tree has a higher likelihood, we move from the current tree to the new one. Repeat the process for every iteration until no better tree can be found within a number of iterations, and the current tree is identified as a local optimal tree. We use a random restart technique after a local optimal tree is found, and try to find as many local optimal as possible within the number of maximum iterations. If multiple local optima are found, we return the tree with the highest likelihood as the optimal GES. The pseudocode of the local search process of LinRace is given in Supplementary Note 1.

### 4.5 Simulating synthetic datasets with TedSim

We use simulated datasets from TedSim which generates paired lineage barcode and gene expression data with ground truth information of the lineage tree. Using the simulated datasets, we are able to benchmark LinRace and other tree reconstruction methods by comparing the reconstructed trees with the ground truth. To simulate with TedSim and test the methods’ potential under various conditions. We use a pre-defined cell state tree (Supplementary Fig. 4) and vary key parameters including mutation rate and the presence of dropout to simulate lineage barcodes of different quality. The selected mutation rate range from 0.05 to 0.4 per target per cell division which covers the realistic ranges of mutation rate in real datasets.

One important advantage of TedSim is that TedSim simulates realistic dropout effects that widely occur in real datasets. In TedSim, when two or more mutation happens at the same cell division, the excision dropout will happen, resulting in deletions of the targets in between. These dropouts can cause a significant decrease in barcode diversity. Comparing the distributions of unique barcodes of real dataset [3] (Supplementary Fig. 1), a simulated dataset with dropout, and a simulated dataset without dropout, we can see that TedSim simulation with dropout is able to generate realistic data with similar negative exponential distribution. In the real dataset of 19019 cells and 2788 unique barcodes, and as mentioned in Results, 1929 of the 2788 barcodes uniquely label one cell; 330 of the 2788 barcodes are shared by two cells. In a simulated dataset of 1024 cells with dropout, 88 cells are uniquely barcoded (no other cell shares the same barcode) and 44 barcodes are shared for 2 cells. This means, 82.8% of the cells share the same barcode with at least two other cells, which is similar to the proportion of 86.4% in the embryo2 dataset in the mouse lineage tracing system. Without dropout, the number of uniquely barcoded cells increases to 336 and 136 barcodes are shared for 2 cells. The proportion of cells that share the same barcode with at least two other cells decreases to 40.6%. These results show that TedSim-generated datasets can benchmark the lineage reconstruction methods fairly under both ideal (no dropout) and realistic (with dropout) situations (Supplementary Fig. 1).

The cell state tree and cell states used for simulation are in Supplementary Note 2. The step size parameter in TedSim is used to sample the cell state tree to obtain discretized cell states from the tree. The step size in our experiments is set to be 0.5 which yields 52 discrete cell states in the simulated datasets. Other static simulation parameters can also be found in Supplementary Note 2.

In the experiment with the C.elegans dataset, TedSim is used to simulate lineage barcodes on the ground truth lineage tree. We also tune the mutation rate from 0.05 to 0.4 per target per cell division and simulate lineage barcode data with and without dropouts. We choose a smaller number of target sites(*Nchar* = 9) considering the size of the lineage(363 cells).

### 4.6 Evaluation metrics for tree reconstruction

To evaluate the accuracy of reconstructed lineage trees, we use two tree comparison metrics: RF distance and Nye similarity. We use the RF.dist function from the phangorn package to calculate the RF distance between reconstructed lineage trees and ground truth trees from simulated datasets. The function returns the ratio of inconsistent splits between the two trees. Therefore, the distance value ranges from 0 and 1, where 0 represents a perfect reconstructed tree and 1 means the worst reconstructed trees where all splits are different from the true lineage. The RF distance is a very strict metric where it only counts as a correct split if only all leaves on the two sides of the splits are consistent for the two trees.

Therefore, we also introduce another metric, Nye Similarity score [22], which can be regarded as an extended version of RF distance. Nye Similarity also compares each split of edge on the two trees, but instead of giving a binary score(0 or 1 for RF distance), it calculates a score based on the similarity between the two splits. Considering each edge *e* in a tree 𝒯, the score $s\left(e_{i},e_{j}\right)$ is for any pair of edges ($e_{i},e_{j}$), $e_{i}\in {𝒯}_{1}$ and $e_{j}\in {𝒯}_{2}$, is obtained by the partition of the leaf nodes. Considering the set of all leaf nodes ℒ, we have:

$$P_{e_{il}}\cup P_{e_{ir}}=P_{e_{jl}}\cup P_{e_{jr}}=ℒ$$

where $P_{e_{il}}$, $P_{e_{ir}}$ are the two disjoint subsets of ℒ by the edge $e_{i}$, and $P_{e_{jl}}$, $P_{e_{jr}}$ are the two disjoint subsets by the edge $e_{j}$. Then, for *r*, *s* = *l*, *r*, the number of leaf nodes shared by the partition can be given as,

$$a_{rs}=\frac{\left|P_{e_{ir}}\cap P_{e_{js}}\right|}{\left|P_{e_{ir}}\cup P_{e_{js}}\right|}$$

For a pair of splits $\left(e_{i},e_{j}\right)$, the score $s\left(e_{i},e_{j}\right)$ is then defined by

$$s\left(i,j\right)=max\left\{min\left\{a_{ll},a_{rr}\right\},min\left\{a_{rl},a_{lr}\right\}\right\}$$

Finally, the Nye Similarity score is derived by maximizing the quantity:

$$\sum_{e_{i}\in {𝒯}_{1}}s\left(e_{i},f\left(e_{i}\right)\right)$$

where $f\left(e_{i}\right)$ is an alignment of edges for the two trees. The quantity is maximized by finding the best alignment of edges *f*.

### 4.7 Running LinRace and baseline algorithms

Here is a summary table of the key packages: lineage inference methods (Cassiopeia, DCLEAR, LinTIMaT), trajectory inference methods (slingshot), data simulation (TedSim) and benchmarking (TreeDist), that are used in this paper:

**Table:** 

Package	Language	Version
Cassiopeia	R	1.0.4
DCLEAR	R	1.0.10
LinTIMaT	Java	-
slingshot	R	2.4.0
TedSim	R	0.0.0.9
TreeDist	R	2.4.1

Major parameters and their settings for LinRace are:

- $\lambda_{1}$ and $\lambda_{2}$, set to be $\lambda_{1}=10$ and $\lambda_{2}=1$ in all tests.
- Number of genes kept after filtering: we keep the top 100 highly variable genes for simulated data and …
- Maximum number of iterations for each local search is 500 in all results.
- Asymmetric division rate $p_{a}$ is set to 0.8 in all tests

Running LinRace-IST also involves the steps of identifying cell states and inferring the cell state tree. *k*-means clustering method is used on the scRNA-seq data to identify cell states and Slingshot is applied to infer the cell state tree, based on the identified cell states. When using simulated data, the number of clusters for *k*-means is set to 7. The same number of clusters is used for the C. elegans data. For the scGESTAULT dataset (ZF1-F3), the number of clusters for *k*-means is set to 20. For the mouse embryo data, we are using annotated cluster ids from the paper.

The parameter settings for baseline methods were mostly based on their default parameters. LinTIMaT has three major parameters: the number of genes *gc*, the number of mutation likelihood iterations *mi*, and the number of combined likelihood iterations *ci*. We set *gc* = 100 when running it on simulated data, the zebrafish dataset, and the mouse embroy dataset. For the C. elegans data, we used *gc* = 93 because the dataset has in total 93 genes. *mi* and *ci* were set to 20000 for both simulated and real datasets.

For DCLEAR, we used the “kmer” mode and set the *k*—mer length to 2 for all datasets, which is the default value for this parameter. We also used default values of other parameters for DCLEAR.

For Cassiopeia, Cassiopeia-greedy and Cassiopeia-hybrid is used for 1024 cells. For the Cassiopeia-hybrid, we set the convergence time limit for the ILP solver to be 1000, the maximum potential graph layer size to be 500, and set a cell cutoff between the top greedy solver and the bottom ILP solver to be 20. For the parameter *indel_priors* (which represents the probabilities of particular indels occurring), we have left it empty.

More detailed descriptions of the parameter settings for running the simulation and the lineage reconstruction methods can be found in Supplementary Note 2.

### 4.8 Reconstruction potential of lineage barcode datasets

Given that lineage tree reconstruction is a difficult problem, we would like to understand the quality of the lineage tracing process using the available barcode data. Given a true lineage tree ${𝒯}^{*}$ and a character matrix *X* (lineage barcodes) of both observed, leaf cells and hidden, ancestral cells (for TedSim datasets, the lineage barcodes of the ancestral nodes are known), we analyze the *reconstruction potential* which can be compared to the Robinson-Foulds distance of a lineage reconstruction method because both metrics are calculated based on the ratio of the splits on the lineage tree. Considering the fact that the true lineage tree is binary, the idea of the reconstruction potential is: for any given edge that splits the whole dataset into two, at least one mutation is required for this split to be reconstructed by any lineage reconstruction method. Denote the true lineage tree as ${𝒯}^{*}=\left(V,E\right)$, where *V* represents the cells on the lineage tree. The reconstruction potential $Q_{r}$ is given as follows:

$$Q_{r}\left({𝒯}^{*}\right)=\frac{\sum_{\left(u,v\right)\in E}𝟙\left(X_{u}==X_{v}\right)}{\left\|E\right\|}$$

where 𝟙(x) is the characteristic function which returns 0 if *x* is TRUE; 0 otherwise. $Q_{r}$ calculates the fraction of edges that contain at least a mutation. This value corresponds to the RF distance if an algorithm perfectly reconstructs every mutation event and does not recover any edges without a mutation event.

## Data Availability

LinRace is available at: https://github.com/ZhangLabGT/LinRace. TedSim is a simulator for paired lineage barcode and gene expression data available at: https://github.com/Galaxeee/TedSim. Cassiopeia is an end-to-end pipeline for single-cell lineage tracing experiments available at: https://github.com/YosefLab/Cassiopeia. DCLEAR is an R package for Distance-based Cell LinEAge Reconstruction (DCLEAR) available at: https://github.com/ikwak2/DCLEAR. LinTIMaT is a statistical method for reconstructing lineages from joint CRISPR-Cas9 mutations and single-cell transcriptomic data available at: https://github.com/jessica1338/LinTIMaT.
